# Salivary Antimicrobial Peptides in Early Detection of Periodontitis

**DOI:** 10.3389/fcimb.2015.00099

**Published:** 2015-12-24

**Authors:** Güliz N. Güncü, Dogukan Yilmaz, Eija Könönen, Ulvi K. Gürsoy

**Affiliations:** ^1^Faculty of Dentistry, University of HacettepeAnkara, Turkey; ^2^Faculty of Dentistry, University of Istanbul MedipolIstanbul, Turkey; ^3^Periodontology, Institute of Dentistry, University of TurkuTurku, Finland; ^4^Welfare Division, Oral Health CareTurku, Finland

**Keywords:** saliva, biomarker, periodontitis, antimicrobial peptides

## Abstract

In the pathogenesis of periodontitis, an infection-induced inflammatory disease of the tooth-supporting tissues, there is a complex interaction between the subgingival microbiota and host tissues. A periodontal diagnostic tool for detecting the initiation and progression of the disease, monitoring the response to therapy, or measuring the degree of susceptibility to future disease progression has been of interest for a long time. The value of various enzymes, proteins, and immunoglobulins, which are abundant constituents of saliva, as potential biomarkers has been recognized and extensively investigated for periodontal diseases. Gingival defensins and cathelicidins are small cationic antimicrobial peptides that play an important role in innate immune response. However, their applicability as salivary biomarkers is still under debate. The present review focuses on proteomic biomarkers and antimicrobial peptides, in particular, to be used at early phases of periodontitis.

## Periodontitis: An orchestral masterpiece

Periodontitis, an infection-induced inflammatory disease of the tooth-supporting tissues, is initiated by the formation of pathogenic biofilms at and under the gingival margin. Out of more than 700 resident bacterial species of the oral cavity, about a half can be found in subgingival biofilms in both healthy and diseased sites (Teles et al., 2006). Periodontitis-associated pathogens and their toxins in bacterial biofilms perturb gingival epithelial cells triggering a sequence of inflammatory and immune responses. The initial inflammatory response aims to limit bacterial invasion by promoting the infiltration of neutrophils and macrophages to the site of bacterial challenge. This infiltration is achieved by the secretion of proinflammatory cytokines and chemokines from gingival epithelial cells and fibroblasts (Preshaw and Taylor, 2011). After their migration to inflamed tissues, leukocytes suppress bacterial invasion by their oxygen dependent or independent mechanisms. Subsequently, T- and B-cells emerge to the site of infection and secrete immunoglobulins as an antigen-specific response. If the host defense fails to suppress the level of infection by eliminating pathogens, continuing inflammation finally ends up in alveolar bone destruction (Bartold and Narayanan, 2006; Preshaw and Taylor, 2011). In the course of the disease, a number of matrix metalloproteases (MMPs), MMP-8, MMP-9, and MMP-13, in particular, are produced and activated by host cells in a cascade leading to degradation of gingival tissues and alveolar bone (Sorsa et al., 2004). Osteoclastogenesis is connected to an increased expression of Receptor Activator for Nuclear Factor κB Ligand (RANKL) and a decreased expression of osteoprotegerin in osteoblast cells. A consensus report of the 7th European Workshop on Periodontology recently highlighted interleukin (IL)-1β, IL-6, tumor necrosis factor (TNF)-α, and RANKL as important players in the periodontitis network (Kinane et al., 2011). Therefore, it does not seem an exaggeration to argue that rather than the bacterial infection, the magnitude of the inflammatory response raised against pathogens seems to be the determinant for developing a destructive periodontal disease (Page and Kornman, 1997; Van Dyke, 2007; Silva et al., 2008). Without an adequate therapy, chronic inflammation may result in the destruction in the attachment between the tooth and the gingival tissue, formation of periodontal pockets, alveolar bone loss and, eventually, tooth loss (Darveau, 2010). Yet, this complex interplay between the subgingival microbiota and host tissues is not the same for everyone; several factors, such as smoking, age, systemic disease, and genetic susceptibility, modify the formation and progression of periodontal diseases (Marsh et al., 2011).

## Saliva as a diagnostic fluid

In general, dental clinicians are looking for a diagnostic tool, preferably a non-invasive one, to determine the current status of periodontal disease, to monitor the response to therapy, and to measure the degree of susceptibility to future disease progression (Giannobile et al., 2009). Conventional diagnostic tools, such as probing pocket depths, bleeding on probing, and clinical attachment level, are inadequate to identify patients who are at risk for disease progression (Goodson, 1992). Saliva has a major importance in the maintenance of oral health, and, during the past two decades, it has been considered a potential specimen to detect oral and systemic diseases (Ji and Choi, 2015). Saliva as a diagnostic fluid has been evaluated for detecting caries (Bratthall and Hänsel Petersson, 2005), periodontitis (Christodoulides et al., 2007; Gursoy et al., 2011), oral and breast cancer (Li et al., 2004; Streckfus and Bigler, 2005), and hepatitis (Ohnishi and Daikuhara, 2003).

With the development of different—omics technologies, analysis of saliva has become particularly fascinating, not only in dentistry but also in general medicine (Zhang et al., 2009; Cuevas-Córdoba and Santiago-García, 2014). By analyzing an array of constituents present in saliva, it is possible to estimate the risk of disease onset, to monitor disease progression, and to evaluate therapeutic efficacy of oral infections as well as oropharyngeal lesions (Zhang et al., 2009).

## Salivary biomarkers of periodontitis: What have been found so far?

Specific biomarkers have been identified from saliva, reflecting the three key features of pathogenic processes in periodontal disease, i.e., infection-induced inflammation, collagen degradation, and bone turnover (Zhang et al., 2009). Host- and bacteria-derived enzymes, proteins, and other inflammatory mediators appear to hold great promise as salivary biomarkers for the diagnosis of periodontal disease (Giannobile et al., 2009). In infected periodontal tissues, numerous cytokines are secreted as part of the innate response by resident cells (epithelial cells, fibroblasts) and neutrophils. There is strong evidence to suggest that salivary IL-1β is a relevant biomarker of periodontitis, while no significant association has been found between salivary TNF-α or IL-6 levels and the presence of periodontitis (Gursoy et al., 2009; Ebersole et al., 2013; Taylor, 2014). Host-derived MMPs are considered initiators of the extracellular matrix degradation associated with periodontitis (Sorsa et al., 1990; Uitto et al., 1990). Especially MMP-8, a neutrophil collagenase, has the potential to be used as a biomarker of periodontal destruction. In a number of studies, it has been shown that MMP-8 activity is elevated in saliva of periodontitis patients as compared to their periodontally healthy controls, regardless of study subjects' smoking status (Sorsa et al., 2004; Miller et al., 2006; Gursoy et al., 2010).

## Antimicrobial peptides: Functions and interactions in periodontal tissues

The oral cavity with its various surfaces offers an open environment, which allows a constant exposure of microorganisms to be colonized and, in favorable circumstances, this can lead to disease (Diamond et al., 2008). Bacterial infection in the mouth is resisted by the stratified squamous epithelium, which acts as a mechanical barrier, and saliva provides a mechanical rinsing action (Gorr, 2012). The oral epithelium and saliva are the most central defense systems in the mouth. These two defense systems do not act as passive protection mechanisms, but both of them contain several types of antimicrobial peptides, including histatins, defensins, and hCAP18/LL-37 (De Smet and Contreras, 2005). Antimicrobial peptides are small cationic peptides with a broad spectrum of antimicrobial activity. A complex mixture of over 45 antimicrobial proteins and peptides are found in oral fluids (Denny et al., 2008); of these, 13 are up-regulated in periodontal disease, while 11 are down-regulated. Defensins and hCAP18/LL-37 belong to key components of the mucosal antimicrobial defense (Hosokawa et al., 2006; Gorr, 2012).

Defensins are divided into subfamilies of α- and β-defensins (Table 1). These defensins differ in their cysteine motifs, but share a similar secondary structure, and both of them are rich in cationic residues (Beckloff and Diamond, 2008). Four types of α-defensins [Human Neutrophilic Peptide (HNP) 1-4] are found predominantly in neutrophils (Ganz et al., 1985), whereas human β-defensins (hBDs) are mainly produced by epithelial cells (Gursoy and Könönen, 2012). The number of hBDs has been suggested to be over 20 based on genomic targeting (Lehrer, 2011). Of the four hBDs known so far, hBD 1-3 are expressed and secreted in the human oral cavity (Dale and Krisanaprakornkit, 2001; Vardar-Sengul et al., 2007). The sole human cathelicidin, hCAP18/LL-37, was initially identified in infiltrating neutrophils in the oral cavity (Dale and Krisanaprakornkit, 2001). However, it has been observed in the salivary glands and gingival epithelium as well (Woo et al., 2003; Gursoy et al., 2012).

**Table 1:** **Comparisons between human β-defensins (hBDs) and human α-defensins (human neutrophil peptides, HNPs)**.

	**Expressed and secreted by**	**Associations with common oral diseases**	**Activation in periodontal tissues**	**Regulatory effects on other host-cells and tissues**
Human β-defensins (hBDs)	Epithelial cells	Caries and periodontitis, oral cancers	hBD-1 is secreted constitutively, while infection and inflammation influence the secretions of hBD-2 and hBD-3	Chemoattraction of dendritic and T cells, macrophages; wound healing in epithelium
Human α-defensins (HNPs)	Neutrophils	Periodontitis, oral cancers	Synthesized in promyelocytes and myelocytes as proHNPs and stored as mature HNPs in azurophil granules before they reach to periodontal tissues	Epithelial cell viability, adhesion, spread

Antimicrobial peptides play a major role in the innate host defense. HNPs, hCAP18/LL-37, and hBDs exhibit a broad-spectrum antimicrobial activity against Gram-positive and -negative bacteria, fungi, and enveloped viruses (Gomes Pde and Fernandes, 2010). Like HNPs, hBDs are considered to exert their antibacterial effect by permeabilizing the bacterial cellular membrane. In addition to their direct antimicrobial activity, both HNPs and hBDs exhibit numerous other biological activities (Dommisch and Jepsen, 2015). HNP 1-3 and hBD 1-3 have a selective chemotactic activity for a variety of host defense cells like immature dendritic cells and mast cells (Soruri et al., 2007). They function as both proinflammatory and anti-inflammatory agents in the periodontal disease pathogenesis (Bowdish et al., 2006). Besides their antimicrobial and immune regulatory functions, hBDs contribute to the healing process of wounds (Niyonsaba et al., 2007) and HNP-1 regulates epithelial cell adhesion and spread (Gursoy et al., 2013). While initially isolated as an antimicrobial peptide, hCAP18/LL-37 has been proposed to play additional roles in inflammation. hCAP18/LL-37 demonstrates a chemotactic activity for neutrophils, monocytes, and some T-cells. Furthermore, hCAP18/LL-37 affects dendritic cell maturation (Kai-Larsen and Agerberth, 2008). Taken together, these multiple activities of antimicrobial peptides suggest that they play an important, multifunctional role in host defense (Gorr and Abdolhosseini, 2011).

It is considered that HNPs, hBDs, and hCAP18/LL-37 have the same function in health and disease, but in a coordinated manner. Periodontal infection and inflammation is thought to affect the expression of each antimicrobial peptide (Gursoy and Könönen, 2012). Up-regulated expression of hBDs has been demonstrated in infections, inflammatory stimulations, and keratinocyte differentiation. In non-inflamed gingival tissues, both hBD-1 and hBD-2 are expressed; their levels are highest at gingival margin close to dental plaque (Yilmaz et al., 2015). During the inflammatory state, these peptides are expressed also in the sulcular epithelium (Dale and Krisanaprakornkit, 2001). The expression of hBD-3 is primarily located in the basal layer of healthy gingival tissues, but it is extended toward superficial layers of the gingival epithelium in periodontitis (Lu et al., 2005; Yilmaz et al., 2015). Although a number of studies have described expression levels and localizations of hCAP18/LL-37, hBDs, and HNPs in healthy and inflamed gingival tissues (Krisanaprakornkit et al., 2000; Dale and Krisanaprakornkit, 2001; Dommisch et al., 2005; Hosokawa et al., 2006; Kuula et al., 2008; Brancatisano et al., 2011; Yilmaz et al., 2015), their relation to the initiation and progression of periodontal disease is still poorly understood.

## Interactions between saliva and antimicrobial peptides: Road to biomarkers

The main sources of antimicrobial peptides in the oral cavity are the gingival epithelium and neutrophils, although salivary glands also secrete some amounts of defensins and hCAP18/LL-37 (Mathews et al., 1999; Mizukawa et al., 1999). By using high performance liquid chromatography, it has been demonstrated that healthy adults have a mean value of 0.5–0.9 mg/ml HNP-1 in whole saliva (Goebel et al., 2000). As a large number of neutrophils continuously enter the oral cavity through the junctional epithelium, it is possible that HNPs are mainly derived from these neutrophils. Immunohistochemical staining for HNP 1-3 showed the presence of peptides in parts of the ductal cells in submandibular glands and in minor salivary glands, while no HNP was detected in an individual major salivary gland (Mizukawa et al., 1999). This indicates that ductal cells can be a source of HNPs in saliva (Abiko and Saitoh, 2007). The sources of hBDs in saliva are assumed to be the oral epithelium and salivary glands. hBD-1, -2, and -3 mRNA have been detected in salivary glands, including the parotid, submandibular, and minor glands, as well as the oral epithelium (Bonass et al., 1999; Dunsche et al., 2001). The mean concentrations of hBD-1 and -2 in whole saliva of healthy subjects are around 150 ng/ml (Mathews et al., 1999), while that of hBD-3 is about 730 ng/ml (Abiko and Saitoh, 2007). In humans, hCAP18/LL-37 is mainly secreted by neutrophils and it is present in saliva at concentrations of 0.14–3 μg/ml (Gorr, 2009).

Saliva carries a significant amount of antimicrobial peptides as part of its defense mechanism but also impairs their antimicrobial functions. As an example, saliva can reduce the antibacterial activities of hBD 1-3 and hCAP18/LL-37 by 20–50% in *in vitro* conditions (Mineshiba et al., 2003). This *in vitro* effect is generally explained by the salt concentration of saliva. However, this is probably unlikely because of the low salt concentrations in saliva. Moreover, hBD activity in saliva may get affected by proteases and redox enzymes. On the one hand, proteases, at least in *in vitro* conditions, affect the activity and concentration of antimicrobial peptides (Kuula et al., 2008), thereby may reduce their value to be used as salivary biomarkers of periodontal disease. On the other hand, defensins are reduced by thioredoxin reductases to their active forms. For instance, glutaredoxin can reduce hBD-1 to its antibacterial form (Jaeger et al., 2013). The activation or inactivation by other proteins in saliva can have a significant effect on the use of antimicrobial peptides as biomarkers, since a selected method for analysis may detect only one form of the peptide, depending on the antibody chosen. Therefore, interactions of antimicrobial peptides with other proteins in saliva should be thoroughly analyzed (Wilson et al., 1999).

## Antimicrobial peptides as salivary biomarkers: How much evidence do we have?

Although, the levels of single markers in saliva can be statistically distinguished between subjects with and without periodontitis, the large variation in their values between individuals make a prospective assignment difficult (Miller et al., 2010). Antimicrobial peptides are typically expressed in response to oral bacteria or bacterial toxins, which makes them suitable biomarkers for the diagnosis of periodontal disease (Gorr, 2009; Gorr and Abdolhosseini, 2011). Information on the association between salivary antimicrobial peptide concentrations and periodontal disease status is limited. Pereira et al. (2013) studied salivary levels of hBD-2 in 31 chronic periodontitis and 27 gingivitis patients, compared to 31 periodontally healthy controls, and detected elevated hBD-2 levels in chronic periodontitis patients. No relationship between the frequency of examined periodontopathogens and hBD-2 protein concentrations was found. Salazar et al. (2013) examined 20 periodontally healthy and 20 diseased subjects to identify periodontitis-associated changes in the proteome of the whole saliva. Twenty proteins, including HNP-1, were elevated in periodontitis patients in comparison to their controls (Salazar et al., 2013). It is important to note that peptide concentrations can be significantly diluted in saliva and, therefore, much lower than those in periodontal pockets and gingival tissues (Gorr, 2012). Salivary LL-37 concentrations have been demonstrated to correlate to periodontal tissue destruction in subjects with chronic periodontitis (Takeuchi et al., 2012).

Advances in genomic technologies offer hitherto unprecedented observations on complex human diseases. To date, however, there is only one study by Jaradat et al. (2013) where associations between the genomic copy number of hBD-2 and periodontitis are evaluated. According to their results, there is an association between decreased hBD-2 genomic copy numbers and severity periodontitis. With increasing information, it may be possible to avoid some of the limitations that currently exist in the use of gingival defensins as biomarkers of periodontitis. Moreover, the outcomes of genomic research would help in understanding clinically distinct diseases, for example Crohn's disease, and periodontitis, with a view on their shared molecular targets, such as hBD-2 (Keskin et al., 2015).

## Things to consider

**Figure 1:**
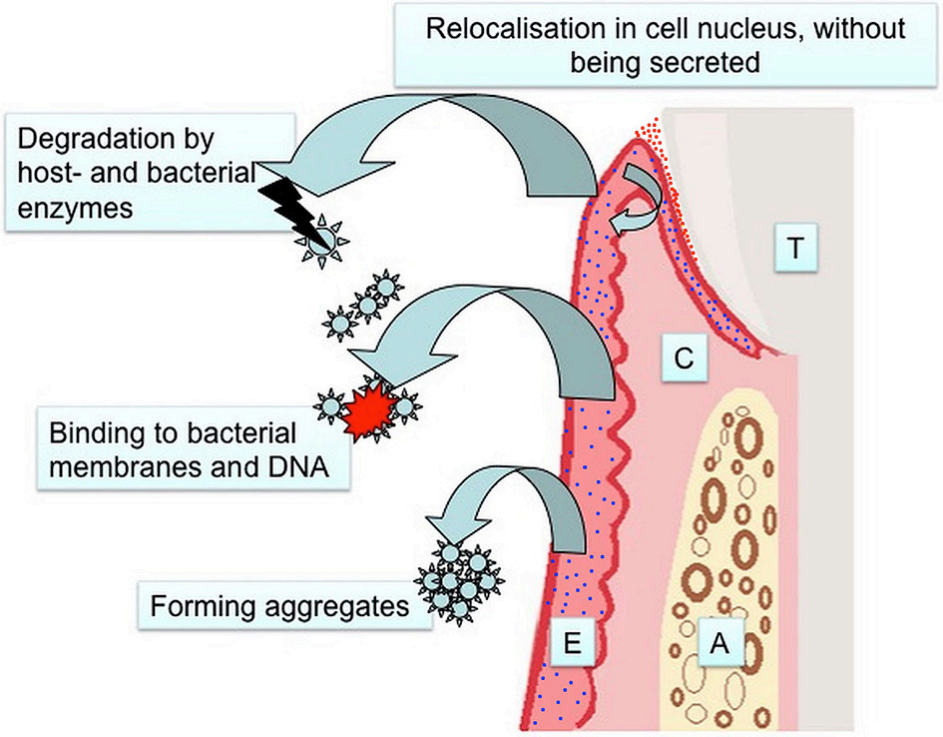
**Limitations in the use of antimicrobial defensins as salivary biomarkers of periodontitis (T, Tooth; C, Connective Tissue; A, Alveolar Bone; E, Epithelium; red dots, α-defensins; blue dots, β-defensins)**.

In this review, we evaluated the evidence on salivary antimicrobial peptides as biomarkers of periodontitis. These small peptides form the initial tissue response against infection and thus could function as an early diagnostic marker of periodontitis. However, in the use of antimicrobial peptides as biomarkers of periodontitis there are significant limitations to consider, and the majority of these limitations are not fully characterized (Figure 1). Firstly, antimicrobial peptides can aggregate in a concentration dependent manner (Brogden, 2005), and this may weaken the sensitivity of test methods, such as an enzyme-linked immunoassay (ELISA). It is also possible that host-derived and bacterial enzymes degrade antimicrobial peptides, decreasing the sensitivity of the methods depending on the antibody of choice. Further, binding to bacterial lipopolysaccharides and DNA may force salivary antimicrobials to accumulate in the pellet at the initial centrifugation of the sample. Finally, some antimicrobial peptides do not get out of the tissue, but accumulate in the cell cytoplasm and cell nucleus as recently described by Yilmaz et al. (2015). They may still be protective against invading bacteria, however, their accumulation tendency affects their concentrations in saliva that will not reflect the degree of inflammation.

## Conclusion

Despite the limitations, it can be concluded that salivary antimicrobial peptides have potential to be considered as early markers of periodontitis. The entire human salivary proteome was reported by a consortium of three research groups, revealing that 1166 proteins are present in human saliva (Denny et al., 2008). In the pathogenesis of periodontitis, antimicrobial peptides cooperate with other inflammatory proteins and regulate distinct inflammatory pathways. Thus, a combinational approach, in which antimicrobial peptides are measured together with their activators or target proteins, will increase their value as diagnostic biomarkers. This aim may be achieved by newly developed omics technologies.

## Author contributions

All authors contributed to the preparation of the manuscript.

### Conflict of interest statement

The authors declare that the research was conducted in the absence of any commercial or financial relationships that could be construed as a potential conflict of interest.
